# Acoustic Performance of Stress Gradient-Induced Deflection of Triangular Unimorphic/Bimorphic Cantilevers for MEMS Applications

**DOI:** 10.3390/ma16052129

**Published:** 2023-03-06

**Authors:** Ning-Hsiu Yuan, Chih-Chia Chen, Yiin-Kuen Fuh, Tomi T. Li

**Affiliations:** Department of Mechanical Engineering, National Central University, Taoyuan City 320317, Taiwan

**Keywords:** stress gradient, deflection, aluminum nitride (AlN), MEMS speaker, sound pressure level (SPL), piezoelectric, unimorph, bimorph

## Abstract

This paper reports two piezoelectric materials of lead zirconium titanate (PZT) and aluminum nitride (AlN) used to simulate microelectromechanical system (MEMS) speakers, which inevitably suffered deflections as induced via the stress gradient during the fabrication processes. The main issue is the vibrated deflection from the diaphragm that influences the sound pressure level (SPL) of MEMS speakers. To comprehend the correlation between the geometry of the diaphragm and vibration deflection in cantilevers with the same condition of activated voltage and frequency, we compared four types of geometries of cantilevers including square, hexagon, octagon, and decagon in triangular membranes with unimorphic and bimorphic composition by utilizing finite element method (FEM) for physical and structural analyses. The size of different geometric speakers did not exceed 10.39 mm^2^; the simulation results reveal that under the same condition of activated voltage, the associated acoustic performance, such as SPL for AlN, is in good comparison with the simulation results of the published literature. These FEM simulation results of different types of cantilever geometries provide a methodology design toward practical applications of piezoelectric MEMS speakers in the acoustic performance of stress gradient-induced deflection in triangular bimorphic membranes.

## 1. Introduction

The conventional speaker generates sound through a magnetic mechanism with a coil and then pushes the air through the vibration of the cone. The MEMS speaker is a device that uses microelectromechanical technology to generate audio. The advantage of the MEMS speaker is excellent audio transparency and high fidelity [1]. It is an integrated piezoelectric element technology in which the structure covers the driver, the actuator, and the piezoelectric material layer. The MEMS microspeaker offers tremendous usage in smartphones, the internet of things (IoT), hearing aids, wearable devices, laptops, and in-ear applications [2,3].

Lead zirconate titanate (PZT), aluminum nitride (AlN), and zinc oxide (ZnO) are widely used for piezoelectric materials [4,5,6]. This research focuses on the comparison between PZT and AlN materials with triangular unimorphic and bimorphic cantilever structures including square, hexagonal, octagonal, and decagonal geometries for piezoelectric MEMS speaker applications. The piezoelectric properties of PZT materials are better than those of AlN materials, and PZT films are widely used in optoelectronics, microelectronics, microelectromechanical systems, and integrated optics. Compared with the piezoelectric PZT film, AlN is an important nitride in the III–V group with a wurtzite structure that exhibits high structural stability [7,8]. In addition, AlN film has the advantage with lower piezoelectric response in which the sound wave velocity of AlN film is higher, meaning that AlN film can be used to make high-frequency filters such as GHz and high-frequency resonance in devices. Although PZT thin film materials have a high dielectric constant, coupling coefficient, lateral piezoelectric coefficient, and vertical piezoelectric coefficient, acoustic wave velocity is low; thus, the PZT materials have poor compatibility. The proportion of individual components and piezoelectric properties are affected by factors such as crystalline orientation, the distribution ratio of components, and particle size. It is difficult to repeatedly produce high-quality PZT thin films. The AlN thin film has better CMOS compatibility and a mature fabrication process [9,10]. In contrast, the fabrication process of PZT is less mature, and the lead content is not environmentally friendly. Therefore, AlN is mainly simulated and discussed in this work.

Traditionally, an important interest in the device design of MEMS speaker is the performance evaluation. Surrounded by various practicable managing factors, residual stress is a major issue directly affecting the structure deflection and performance variations [11]. Consequently, the characterization of residual stress in MEMS structures is a significant part in various aspects. Residual stresses in thin film can be regarded as the combination of a uniform stress and a stress gradient across the thickness [12]. During the release process by removing the sacrificial layer, the phenomena of deflection generated by residual stress and a stress gradient will influence the speaker performance.

The sound pressure level (SPL) of microelectromechanical systems (MEMS) speakers with and without stress gradients in physical vapor deposition (PVD) was compared in this study and it was found that the SPL without stress gradients had the best performance. The slope of the stress gradient was also found to impact the amount of buckling that occurs on the wafer, which can affect the wafer yield and the performance of the SPL [13]. To better understand this phenomenon, the electric field distribution on the surface of the wafer in a PVD chamber was simulated using COMSOL software. One of the factors that can contribute to non-uniform wafer thickness is the uneven distribution of electric field. A non-uniform distribution of the electric field was found to lead to non-uniform deposition of the wafer, resulting in a stress gradient on the wafer surface that increases from the center to the edge. Different geometric structures of the MEMS speaker membranes were also observed to have varying degrees of deflection under the same stress gradient, with more circular structures deflecting less and thus being less affected by the stress gradient. By controlling the stress gradient, it was found that it is possible to improve the wafer yield. The aim of this study is to achieve an acoustic performance of stress gradient-induced deflection of triangular unimorphic/bimorphic cantilevers for practical applications of piezoelectric MEMS speakers.

## 2. Simulation Set-Up, Speaker Design and Geometry

### 2.1. Simulation Set-Up: Parameters

**Figure 1:**
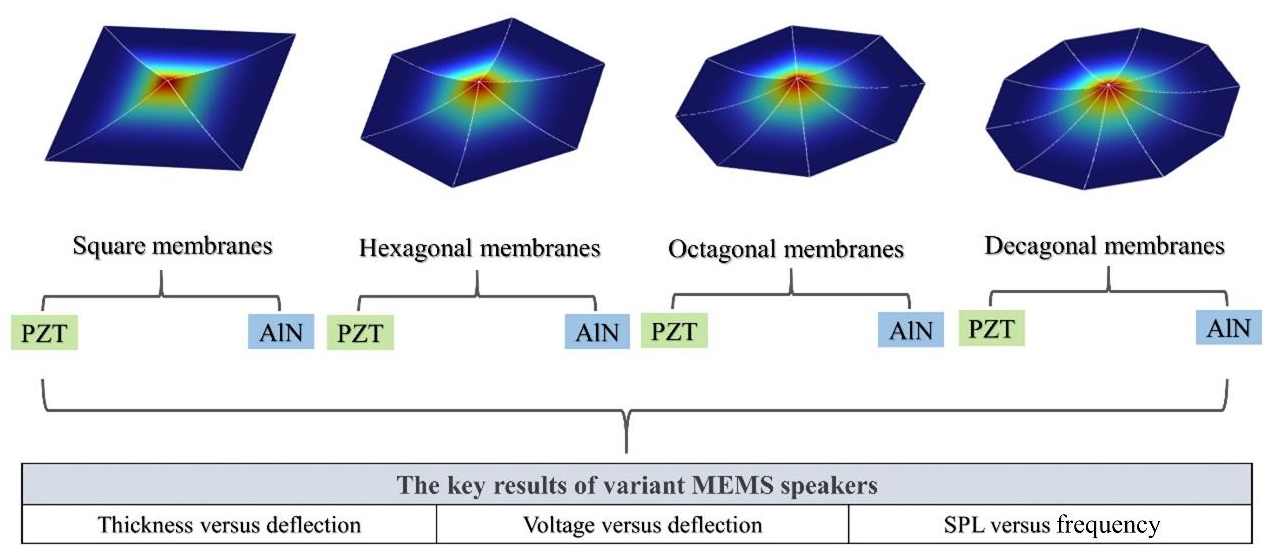
Illustration of the various cantilever geometries of MEMS speakers and key results of variant MEMS speakers.

We designed unimorphic and bimorphic loudspeakers with four different cantilever geometries including square, hexagon, octagon, and decagon in triangular membranes, and utilized COMSOL Multiphysics (COMSOL, Inc., Burlington, MA 01803, USA, 6.0) or simulation in the study of physical and structural properties of piezoelectric devices. Figure 1 shows the simulation parameters with associated key results on variants of MEMS speaker designs including the thickness in active layer, the piezoelectric materials in active layer (PZT and AlN), the molybdenum (Mo) electrode material, the driving voltage, and the SPL between the unimorphic and bimorphic structures. The key parametric variant-associated results will be discussed in the following sections as well as in the Results and Discussion.

### 2.2. Speaker Geometry

**Figure 2:**
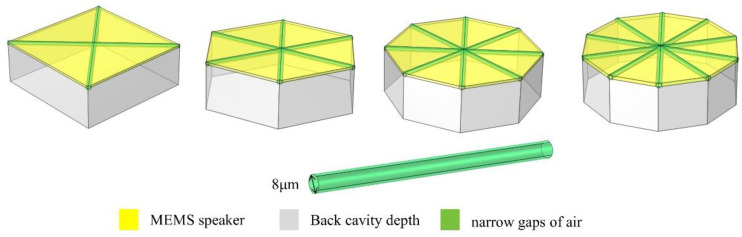
Diagram of the bimorphic MEMS speakers with various cantilevers: square, hexagonal, octagonal, and decagonal geometries.

**Figure 3:**
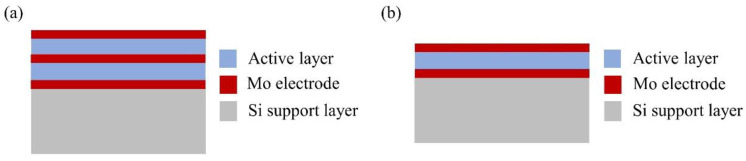
Cross-section of the unimorphic and bimorphic speakers. (**a**) Mo is used as the bottom, middle, and upper electrode in a bimorph device. (**b**) Mo is used as the bottom and upper electrode in a unimorphic device.

The unimorphic and bimorphic structures have an active area of about 10.39 mm^2^ with an air gap of 8 μm, and the active layer is composed of PZT and AlN piezoelectric materials [14]. Each active layer is connected to a layer of electrodes, and the proposed structure is illustrated in Figure 2. Unimorph and bimorph cross-sectional structures can be realized as shown in Figure 3. For a bimorphic device, molybdenum (Mo) is used as the bottom, middle, and the upper electrode, see Figure 3a. For a unimorphic device, Mo is used as the bottom and upper electrode, see Figure 3b.

### 2.3. Speaker Design

Figure 3 shows the schematic cross-sections of the unimorphic and bimorphic speakers. Wang et al. developed a MEMS loudspeaker with a side length of 2 mm and six diaphragms [15]. A remarkable SPL improvement was obtained at low frequency compared to other existing MEMS speakers [15,16,17,18,19]. The simulation size of the different cantilevers in a unimorphic structure is set to 10.39 mm^2^ and the air gap width is ~8 μm. Lang et al. designed and fabricated a bimorphic diaphragm with dimensions of 1.4 × 1.4 mm^2^. The purpose is to reduce the area of the loudspeaker while achieving a high sound pressure level of the loudspeaker. Thus, the area of the loudspeaker diaphragm is set to be 1.4 × 1.4 (1.96) mm^2^ [9]. The resonant frequency is fixed at ~10 kHz. A resonant frequency that is up to 10 kHz can give the loudspeaker better high-frequency performance and wider bandwidth. The thickness of the AlN active films is fixed at 1 μm to guarantee the quality of the films. This paper reports on the simulation of MEMS loudspeaker diaphragms with four different geometric cantilever structures. The area is about 10.39 mm^2^, and the thickness of each unimorph and bimorph active layer is 500 nm for both AlN and PZT. The thickness of Mo is 25 nm as an electrode, and the thickness of Si is 5 µm as a support layer. For simulation purposes, the symmetry condition requires two halves adjacent to each device and a single air gap. The driving voltage between the top electrode and bottom electrode ranges from 2 V to 20 V. The cantilever geometries are separated by narrowing air gaps, and the full thermo-viscous acoustic formulation is used to simulate the acoustic behavior. The simulated results are based on the physical conditions of solid mechanics, electrostatics, and pressure/thermo-viscous acoustics. Table 1 lists the materials properties (AlN, Si, Mo, SiO_2_, and PZT) that we use for simulation.

## 3. Results and Discussion

### 3.1. Film Thickness versus Deflection

**Figure 4:**
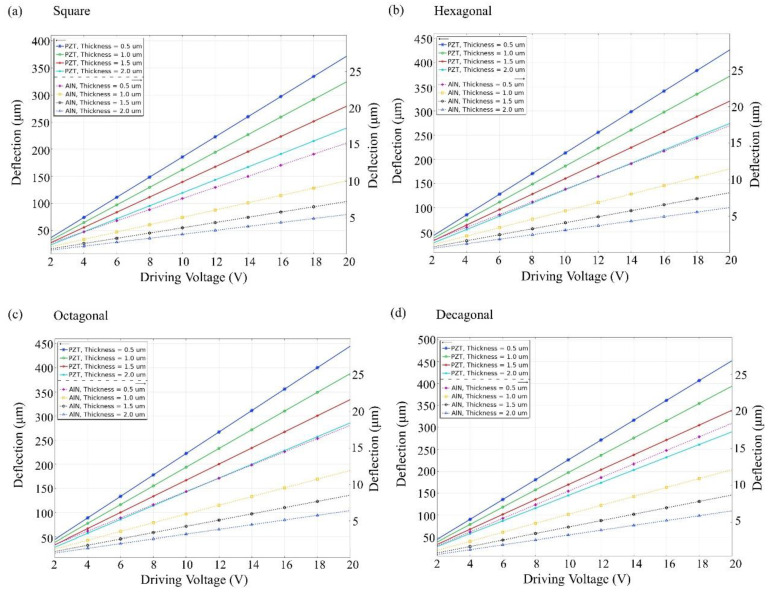
Unimorph active layer on silicon support layer 5 (µm). Maximum deflection amplitude vs. driving voltage at different thicknesses for (**a**) square, (**b**) hexagonal, (**c**) octagonal, and (**d**) decagonal membranes.

**Figure 5:**
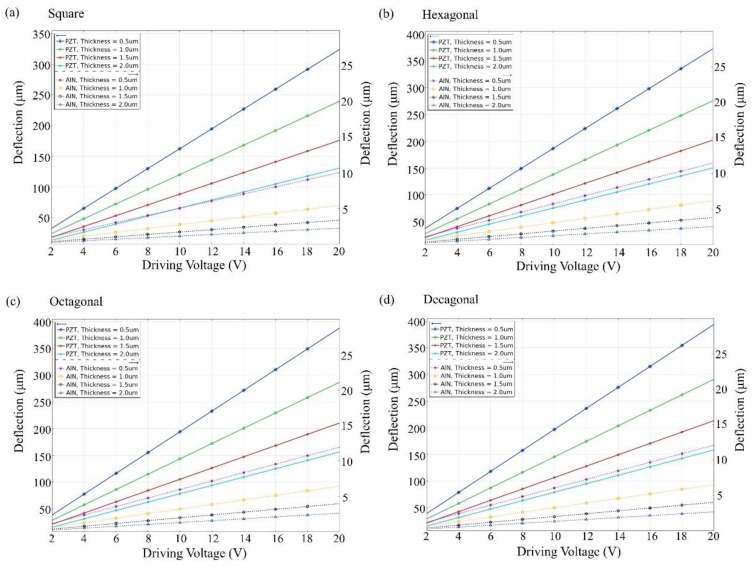
Bimorph active layer on silicon support layer 5 (µm). Maximum deflection amplitude vs. driving voltage at different thicknesses for (**a**) square, (**b**) hexagonal, (**c**) octagonal, and (**d**) decagonal membranes.

Figure 4 and Figure 5 show that the unimorph and bimorph active layers are applied with different voltages at different thicknesses. For the unimorph active layer with different thicknesses, the maximum deflection value of the PZT material can reach ~450 µm and the maximum deflection value of the AlN material can reach ~18 µm. For the bimorph active layer with different thicknesses, the maximum deflection of the PZT material can reach ~390 μm and the maximum deflection of the AlN material can reach ~12 μm. The deflection of the PZT material is much higher than that of the AlN material. The result shows the decagonal active layer of the unimorph active layer has the largest deflection, and the octagonal and decagonal active layers of the bimorph layer show less deflection. The active layer has thicknesses of 0.5, 1.0, 1.5, and 2.0 µm, and a thickness of 0.5 µm is much better than the others. As shown in Figure 4 and Figure 5, the largest deflection is achieved at a layer thickness of 0.5 µm. Therefore, the thickness of the unimorph and bimorph active layers in this study was set at 500 nm (0.5 µm) for both AlN and PZT.

### 3.2. Driving Voltage versus Deflection

**Figure 6:**
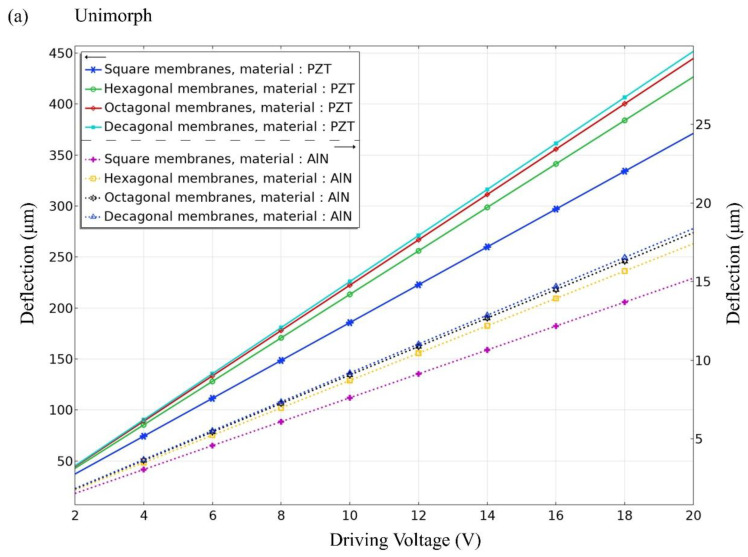

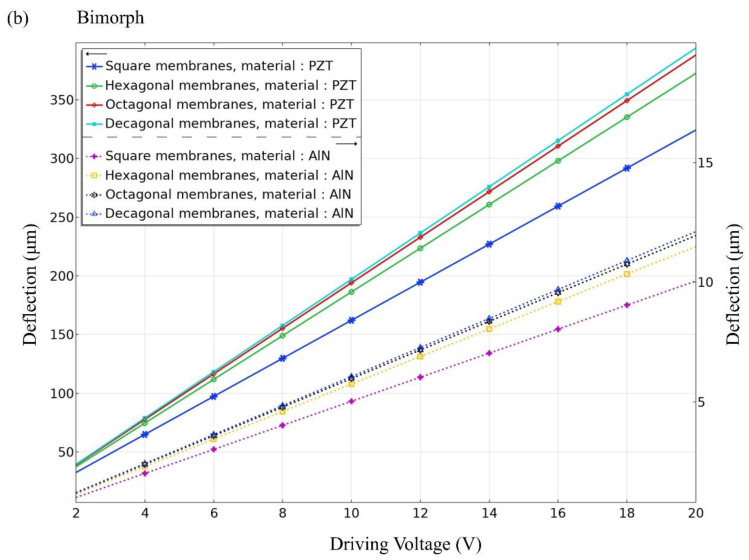
At the same driving voltage, the active layer composed of PZT shows higher sensitivity than AlN. It also proves that the cantilever geometry of the diaphragm can improve the performance of the loudspeaker for deflection amplitude vs. driving voltage at a fixed thickness of 500 nm: (**a**) unimorphic diaphragm and (**b**) bimorphic diaphragm.

As shown in Figure 6, the unimorph and bimorph active layers with different geometric cantilever structures are subjected to different voltages at a fixed thickness of 500 nm. The specified voltage range is between 2 V and 20 V. From Figure 6, it shows the larger the applied voltage, the larger the deflection. For the four different geometric cantilever structures, the deflection is the largest for decagonal geometry. Figure 6a shows that the deflection of the decagonal PZT active layer can be up to 450 µm and the deflection of the AlN active layer can be up to 18 µmm, both in a unimorphic diaphragm. In Figure 6b, the deflection of the decagonal PZT active layer can be up to 390 µm and the deflection of the AlN active layer can be up to 12 µm in a bimorphic diaphragm. However, AlN piezoelectric material is more compatible with CMOS manufacturing than PZT piezoelectric material and is more environmentally friendly without lead. The fabrication process of AlN film is more mature than that of PZT, therefore AlN is mainly simulated and discussed in this study.

### 3.3. Film Stress Gradient Distribution versus Deflection

In a vacuum plasma chamber, plasma is an ionized gas made up of charged particles and atomic nuclei. The electron density refers to the number of electrons present within a specific volume. An electric field is a potential difference created by charged particles, which can be used to describe the distribution and movement of charges. There is a close relationship between plasma, electron density, and the electric field [20]. The density of plasma is related to the electron density since most of the particles in plasma are electrons. There is also a connection between the electron density and the electric field, as the electric field can impact the movement of electrons. For instance, when the electric field strength increases, the speed of electron movement increases, leading to an increase in electron density. At the same time, electron density can also affect the electric field, as the presence of electrons causes a change in charge distribution, which in turn affects the strength of the electric field. In general, in a vacuum plasma chamber, plasma, electron density, and the electric field are interrelated and have a close connection with each other. During the process of depositing thin films, charged particles in the plasma can deposit onto the substrate through the electric field. The distribution of the electric field on the substrate is influenced by various factors, including the material properties of the substrate, plasma characteristics, and chamber design. By appropriately adjusting these factors, the distribution of the electric field can be controlled, which in turn affects the mass, thickness, and chemical composition of the thin film.

**Figure 7:**
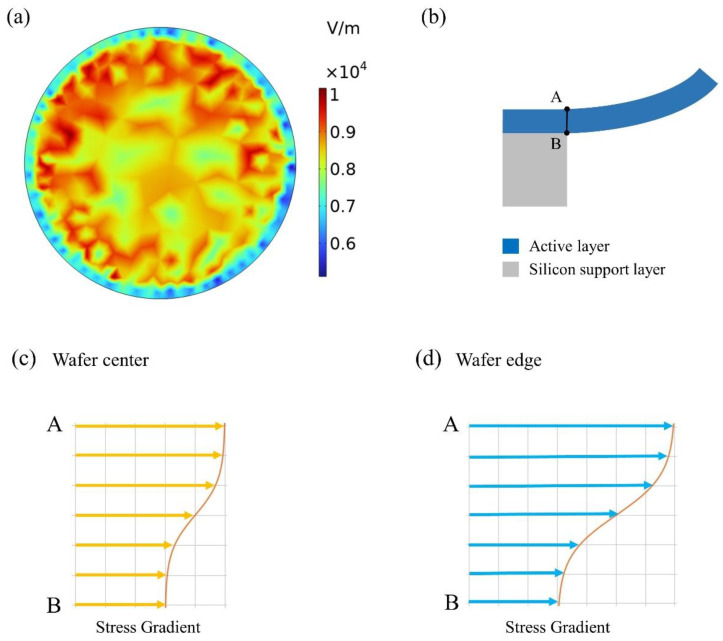
(**a**) Electric field distribution induced by plasma on wafer surface of AlN layers. (**b**) Cross-section AB of single diaphragm. (**c**) Stress gradient at wafer center. (**d**) Stress gradient at wafer edge.

In PVD processes, in order to ensure sufficient thickness distribution at the edge of the wafer, we face a great challenge to supply larger plasma intensity at its edge [21]. If we ignore the importance of the plasma parameter, this leads to an increase in the ion bombardment at the wafer edges which takes place when increasing the value of the residual stress in conventional DC-pulsed processes. The close relationship between plasma, electron density, and electric field in a vacuum plasma chamber is evident. An inhomogeneous plasma can lead to an inhomogeneous electric field distribution (Figure 7a) that produces different stress distributions in AlN layers, from which emerge the different stress gradients from the bottom to top surface of the AlN layer (see Figure 7c,d). According to the fabrication process, the residual stress and stress gradient will cause either tensile or compressive stress through the AlN layer thickness. Figure 7b schematically shows the cross-section AB of a single diaphragm. As shown in Figure 7c,d, the difference of the local stress between the bottom and top surface at the edge area can be distinguished, where the stress at the edge area is much larger than that at the center area. By utilizing FEM, the electric field on wafer surface was simulated, which demonstrated the non-uniform distribution of the electric field on wafer surface was induced by plasma. The increase in the ion bombardment that occurred at wafer edge brings out the different stress gradients from the wafer center to wafer edge.

As a matter of fact, according to the study of Lang et al., the performance of SPL within FEM-simulated results generated by a bimorphic structure will be better than a unimorphic structure [9]. Consequently, we utilize the bimorphic structure for further research.

**Figure 8:**
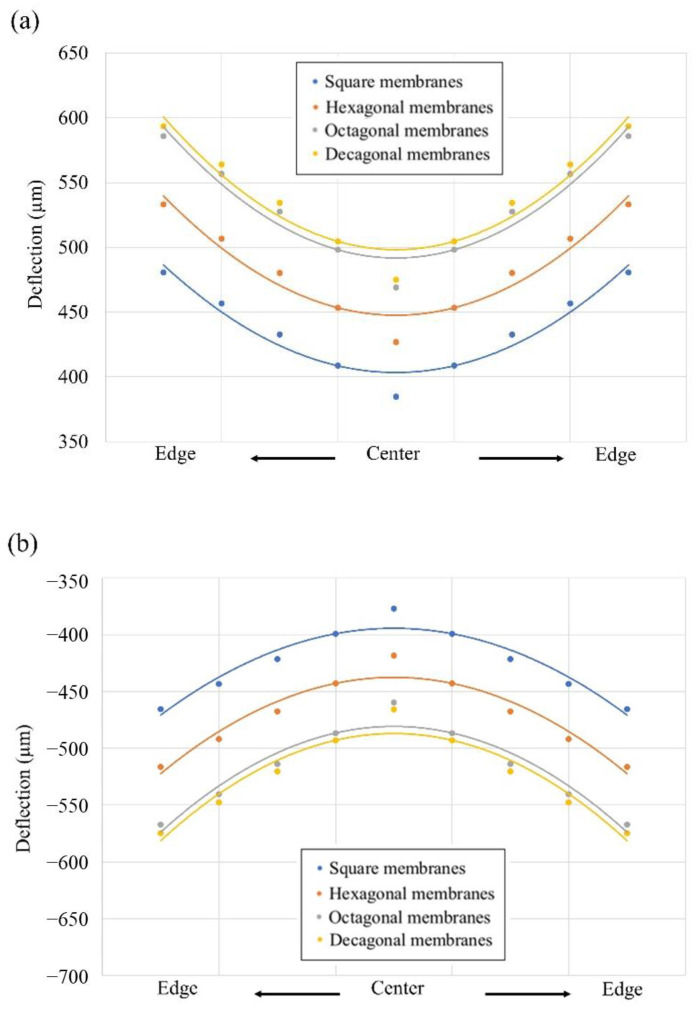
(**a**) Positive curvature of deflection in membranes as a tensile stress. (**b**) Negative curvature of deflection in membranes as a compressive stress.

In most of the designs, stress gradient is the major problem from the results in positive or negative beam curvature, therefore, it is important to reduce the influence of the stress gradient. The increasing residual stress becomes more non-identical as the distance of device location starts from the wafer center [22,23,24]. Moreover, we checked it with FEM analysis to determine if this could be due to residual stress and stress gradient. Table 2 lists the stress positions in AlN bimorphic layers. As shown in Figure 8, for these simulations with the increasing residual stress and different stress gradients, the triangular cantilever membrane with the increase in deflection amplitude is located starting at the wafer center toward the wafer edge. Since the residual stress $\sigma_{2}\left(\mathrm{top}\;\mathrm{surface}\;A\right)>\sigma_{1}\left(\mathrm{bottom}\;\mathrm{surface}\;B\right)$ in tensile stress, the direction of deflection points is up (positive curvature) (Figure 8a); if the residual stress $\sigma_{2}\left(\mathrm{top}\;\mathrm{surface}\;A\right)<\sigma_{1}\left(\mathrm{bottom}\;\mathrm{surface}\;B\right)$ in compressive stress, the direction of deflection points is down (negative curvature) (Figure 8b) [25].

**Figure 9:**
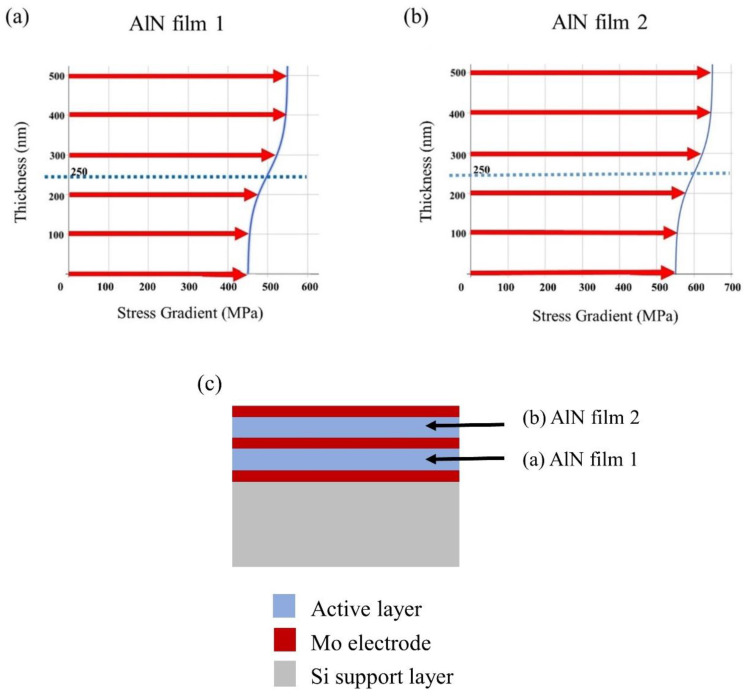
The stress gradient of bimorphic membranes: (**a**) the average stress in AlN film 1, (**b**) the average stress in AlN film 2, and (**c**) schematic cross-section of AlN film 1 and AlN film 2.

During the manufacturing process, film residual stress cannot be eluded but can be minimized by modifying the parameters of the manufacturing process, including the composition selection of film and seed layer material applied for the growth of freely moving parts, i.e., membranes of the MEMS speaker [26,27]. The film residual stress plays a key role in a mechanical structure, which can lead to significant changes in the MEMS speaker. Figure 9a,b presents the residual stress gradients in AlN film 1 near the bottom electrode and AlN film 2 near the upper electrode, respectively. It can be seen that different results can be obtained when the voltage remains the same but the stress gradient is changed. Figure 9a,b shows the variation in the stress gradient of AlN film 1 and film 2 with increasing thickness. The residual stress in average is 500 MPa and 600 MPa, respectively. With the difference in stress gradient coefficient ω (Equation (1) below), the local stress (σ) changes from rapid to slight, and the given stress value range is ±50, i.e., between 450 MPa and 550 MPa. While tuning the stress gradient coefficient ω, the curvature of the stress distribution can be tuned from curve distribution to linear distribution. The curve that approaches the value of the stress gradient seems to be more linear and the deflection is minimal. Figure 9c shows the cross section of the diaphragm which is assembled by Mo (electrode) and AlN (film 1 and film 2).

### 3.4. SPL versus Frequency in Different Cantilever Geometries

The local stress at different heights of the film with respect to the average stress of the film can follow Equation (1) below:

(1) $$\sigma_{local\;stress}=\sigma_{mrs}+\sigma_{∆\sigma }\tanh\left(\frac{Z-h_{m}}{\omega }\right)$$

The film stress gradient can be addressed as a composite material with N layers of AlN films, where $\sigma_{local\;stress}$ (MPa) is the local AlN film residual stress, $\sigma_{mrs}$ (MPa) is mean residual stress, $\sigma_{∆\sigma }$ is the difference of residual stress between upper surface/lower surface and middle surface in thin film, which causes the stress gradient in thin film, Z (µm) is the height of thin film, $h_{m}$ (µm) is the height of middle surface, and ω is the coefficient of stress gradient.

**Figure 10:**
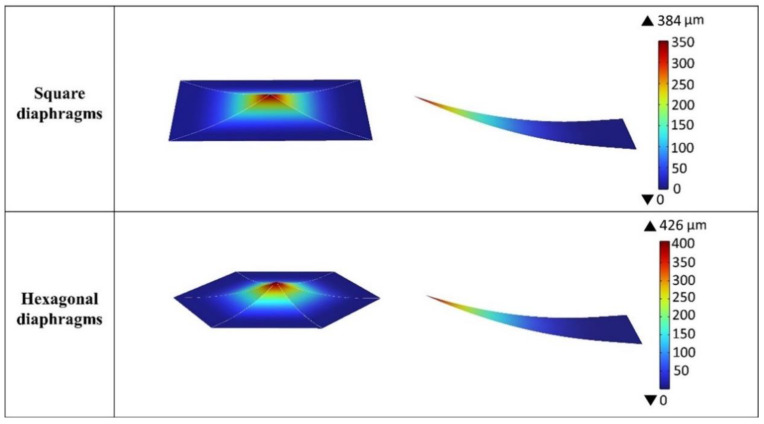

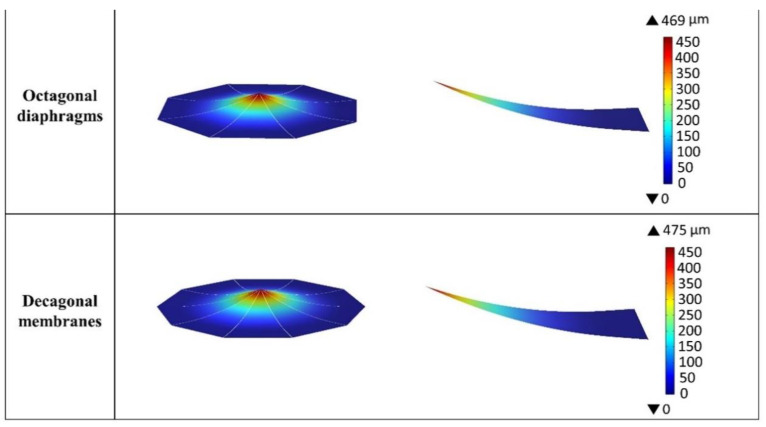
The deflection of the diaphragm after applying stress gradient with different bimorphic cantilever geometries in a triangular membrane.

In the application of stress gradients to different bimorphic cantilever geometries in a triangular membrane, Figure 10 shows the deflection results in the FEM analysis with four different geometries including square, hexagonal, octagonal, and decagonal diaphragms. The deflection for the different cantilever membrane geometries are 384, 426, 469, and 475 µm, respectively, with the same average residual stress and stress gradient. The results from FEM show that the four types of devices have the same area but decreasing the area of a single membrane in a device leads to an increase in deflection.

The speaker is located in an infinite baffle and uses a voltage difference acting between the faces of the piezoelectric material to create vibrations that will propagate as acoustic perturbations. The acting voltage contains a constant component, usually called the bias voltage (DC), and an alternating voltage (AC or perturbation contribution); it follows Equation (2) [28]:

(2) $$V_{0}=V_{DC}+V_{AC}\cos\left(2\pi f\cdot t\right)$$

where *V*_0_ is the terminal voltage, *V_DC_* is the bias voltage, *V_AC_* is the alternating voltage, *f* is the driving frequency, and *t* is the time.

**Figure 11:**
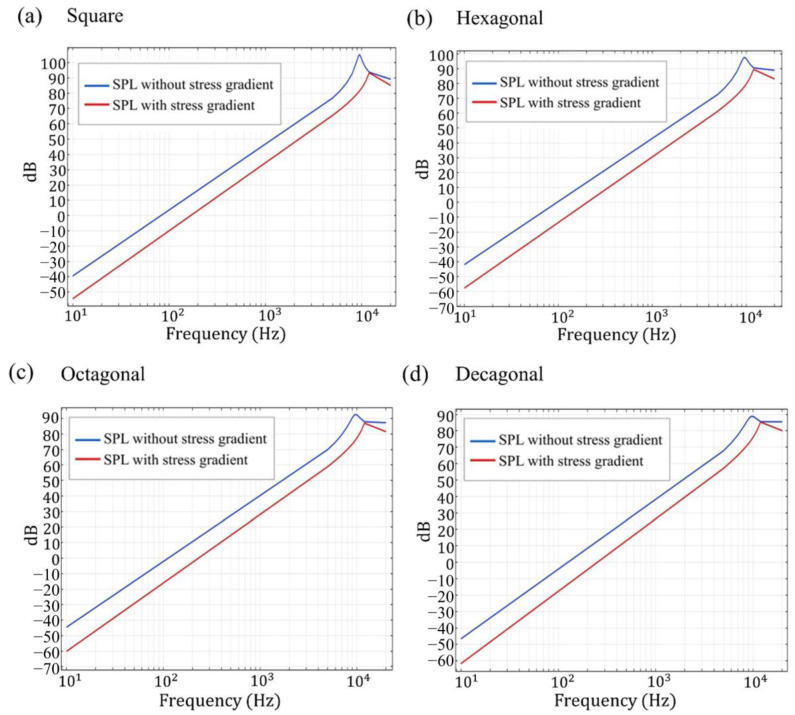
SPL simulation of SPL vs. frequency for (**a**) square, (**b**) hexagonal, (**c**) octagonal, and (**d**) decagonal cantilevers.

During the manufacturing process, the generation of local residual stress cannot be avoided. When we compare the performance of SPL with local residual stress and without local residual stress at 10 V driving voltage, as shown in Figure 11 [29], the result shows that SPL without local residual stress is better than SPL with local residual stress. For the bimorphic loudspeaker, the simulation of SPL in different geometries shows that the highest value of SPL for the square geometry without local residual stress gradually decreases, and the value of SPL for the square geometry is still the highest when local residual stress is present. The second highest value is for the hexagonal geometry, as shown in Table 3. In addition, with the simulated condition of stress gradient, the SPL results show less difference in the decagonal structure between the condition of non-utilizing stress gradient and the condition of utilizing stress gradient. The attenuation of SPL in the decagonal structure is much smaller than the square structure with the condition of stress gradient, which is close to the circumstances of reality.

### 3.5. Literature Results for Comparison

**Figure 12:**
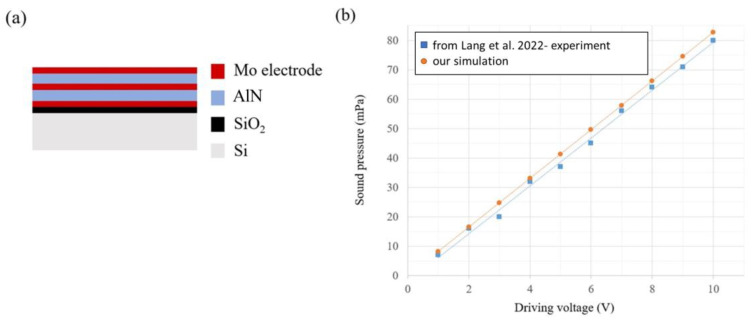
(**a**) MEMS speaker of a bimorph diaphragm on the square cantilever geometry in literature reference [9]. (**b**) The linear relationship between the sound pressure produced by the fabricated bimorph speaker in a square shape (1.4 × 1.4 mm^2^) and the driving voltage.

Most recently, Lang et al. [9] used FEM to simulate the acoustic performance of a bimorph speaker structure with dimensions of 1.4 × 1.4 mm^2^ in a square shape by active layers of 1 µm aluminum nitride (Figure 12a). The simulation results verified the feasibility of the bimorph diaphragm and the improved SPL on the fabricated speakers. The test results showed a good linearity between the sound pressure produced by the fabricated speaker and the driving voltage (see Figure 12b) [9]. Additionally, in order to cross-check the accuracy of our simulation, we compared the simulation results between our simulation model and the reference design [9]. Our FEM model is shown to be reasonably accurate and suitable for this study, as illustrated in detail below. Our simulation results exhibited a strong resemblance to the experimental measurements reported in the literature.

**Figure 13:**
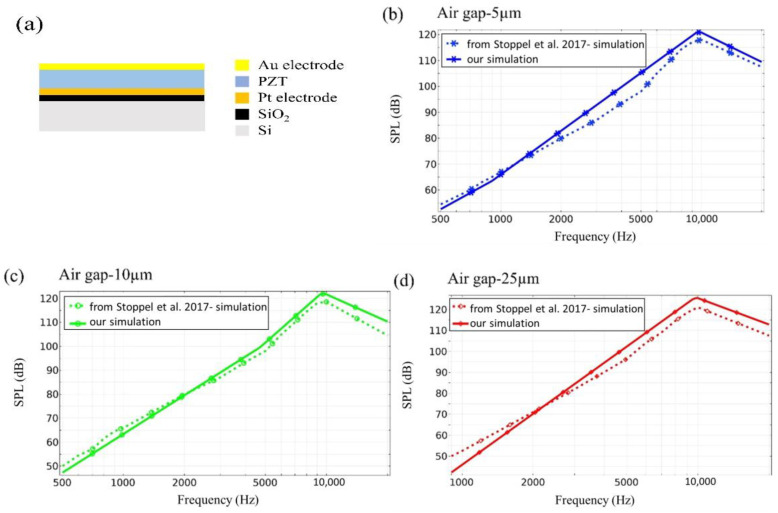
(**a**) MEMS speaker of unimorph diaphragms on the square cantilever geometry in literature reference [6], and simulation results of SPL vs. frequency with different air gaps, (**b**) 5 µm, (**c**) 10 µm, and (**d**) 25 µm, respectively, in the cantilever geometries of unimorph diaphragms.

On the other hand, in order to thoroughly evaluate the simulation model’s accuracy, we performed a comparison between our simulation results and those results presented by Stoppel et al. [6] for simulating the SPL of a speaker structure, which included a 2 µm thick PZT active layer and a 15 µm poly-Si support layer in a unimorph diaphragm with dimensions of 4 × 4 mm^2^ as shown in Figure 13a. Figure 13b–d displays the simulation results in reference [6] under three different air gap conditions of 5 µm, 10 µm, and 25 µm. Our simulation results are slightly different from the reference paper and experimental results due to some setting conditions that were not explicitly mentioned in the paper, such as mesh size, boundary conditions, and back cavity size. However, the differences are very small, indicating that our simulation model settings are almost identical to those of the reference paper, which provides strong evidence of the simulation model’s credibility and accuracy.

**Figure 14:**
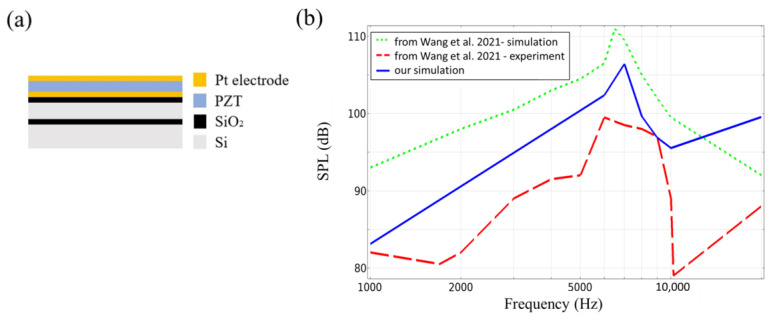
(**a**) MEMS speaker of a unimorph diaphragm on the hexagonal cantilever geometry in literature reference [15]. (**b**) The results of our simulation (blue line) compared with the simulation result (green dotted line) and experimental measurement (red dotted line) in literature reference [15].

Furthermore, based on the reference design of Wang et al. [15], which has a unimorph diaphragm on the hexagonal cantilever geometry with a side length of 2 mm (Figure 14a), we compared our simulation results of the SPL versus frequency with both experimental measurements and simulation results reported in the literature (reference [15]). We found that our simulation results were closer to the experimental measurement curve reported in the literature, while the simulated results in the literature showed significant differences.

## 4. Conclusions

In this study, we aimed to investigate the acoustic performance of triangular unimorphic/bimorphic cantilevers for MEMS applications. To do so, we simulated and analyzed the performance of unimorph and bimorph active layers under various simulation parameters, including geometry, thickness, voltage, and stress gradient, using solid mechanics, electrostatics, and pressure/thermo-viscous acoustics physical conditions. With the residual stress and stress gradient affected by plasma in the PVD process, the deflection form location of wafer center to wafer edge displayed a positive or negative curvature. On the other hand, the simulation shows that the active layer of AlN has the largest deflection at a thickness of 0.5 μm by 20 driving voltage. When the deflection was simulated at a fixed thickness of 0.5 μm with variable voltage, the result of the simulation shows the higher the voltage, the larger the deflection.

In fact, we compared our simulation results with the experimental measurements reported in the literature references [9,15] for both bimorphic square cantilever structures and unimorphic hexagonal cantilever structures. The results demonstrated the accuracy of our simulation, as our simulation results were found to be highly similar to the experimental measurement data reported in the literature.

In our design, while applying driving voltage of 20 V, the speaker resonated at around 10 kHz. Under that resonant frequency, the SPL was from 105 dB to 89 dB, and with the initial deflection condition, the SPL was from 93 dB to 85 dB with the membrane morphology of square to decagon. However, during the fabrication of sputtering, residual stress is inevitable, and the deflection after release is generated by residual stress and stress gradient which are the major factors that affect the SPL of the device. For the bimorphic loudspeaker, the simulation of SPL in different geometries showed the highest value of SPL for the square geometry with and without local residual stress. In addition, with the simulated condition of stress gradient, the SPL results show less difference in decagonal structure between the condition of non-utilizing stress gradient and the condition of utilizing stress gradient. Furthermore, the stress gradient-induced deflection may also consider the non-uniform deflection within one die, i.e., mismatch (the difference between the maximum and minimum deflection) and relevant impact on the acoustic performance.

In future work, we are planning to fabricate bimorphic MEMS speakers with various cantilevers: square, hexagonal, octagonal, and decagonal geometries. For optimizing the thickness and membrane morphology of the diaphragm, the PVD fabrication process to avoid high residual stress and stress gradient, as well as the experimental validation afterwards, will be the first concern in order to attain a better SPL performance. Furthermore, acoustic measurement work will be carried out, including for the performance of microspeakers such as SPL, total harmonic distortion (THD), and sensitivity.

## Figures and Tables

**Table 1:** Material properties.

Material	Young’s Modulus E (GPa)	Poisson’s Ratio	Density ρ (kg/m^3^)	Piezoelectric Coefficient (d_31_) (pm/V)	Dielectric Constant (ε_r_)
AlN	345	0.24	3300	−1.92	9
Si	170	0.28	2329	-	-
Mo	312	0.31	10,200	-	-
SiO_2_	70	0.17	2200	-	-
PZT	63	0.31	7500	−1.80	762.5

**Table 2:** Conditions of AlN stress in AlN bimorphic layers from center position to edge position of wafer.

Residual Stress (MPa)					
**Location**	**Edge**	** 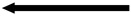 **	**Center**	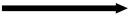	**Edge**
AlN film 2	800		600		800
AlN film 1	600		500		600

**Table 3:** SPL values for different diaphragms.

Geometry of Speakers	SPL (dB) without Stress Gradient	SPL (dB) with Stress Gradient
Square	105	93
Hexagonal	97	89
Octagonal	92	86
Decagonal	89	85

## Data Availability

The data presented in this study are available on request from the corresponding author. The data are not publicly available due to that the device is currently still under development, and therefore, we are unable to provide data at present.
